# Circle RNA circ_0007331 promotes colorectal carcinoma by targeting miR-205-5p/high-mobility group A2 axis

**DOI:** 10.1080/21655979.2022.2051857

**Published:** 2022-04-10

**Authors:** Lihui Wang, Weiming Weng, Shuhui Yang, Shasha Yang, Richang Du

**Affiliations:** a Department of Pathology, Yuebei People’s Hospital, Shaoguan, Guangdong, China; b Department of Gastrointestinal Surgery, Yuebei People’s Hospital, Shaoguan, Guangdong, China

**Keywords:** colorectal cancer, circ_0007331, HMGA2, circle RNA, miR-205-5p

## Abstract

Colorectal cancer (CRC) is a common malignancy of the gastrointestinal tract. CircRNAs have been reported to play regulatory roles in many cancers, including CRC. This study focuses on the role of circ_0007331 in CRC. Differentially expressed circRNAs in CRC were screened using the GEO database. RT-qPCR was used to analyze mRNA expression. StarBase and TargetScan were used to predict targeting relationships and then verified by the dual luciferase reporter assay along with the RNA pull-down assay. CCK-8 as well and transwell assays were used to measure cell viability, migration, and invasion. Protein levels were determined using western blotting. circ_0007331 is expressed more frequently in patients with CRC. The inhibition of circ_0007331 expression reduced the viability, colony formation, migration, and invasion of CRC cells. However, inhibition of miR-205-5p or elevation of high-mobility group A2 (HMGA2) can reverse the function of inhibited circ_0007331 in tumor cells. This study demonstrated that the circ_0007331/miR-205-5p/HMGA2 axis promotes CRC development. Thus, circ_0007331 may be a potential biomarker for CRC.

## Graphical Abstract

**Figure:**
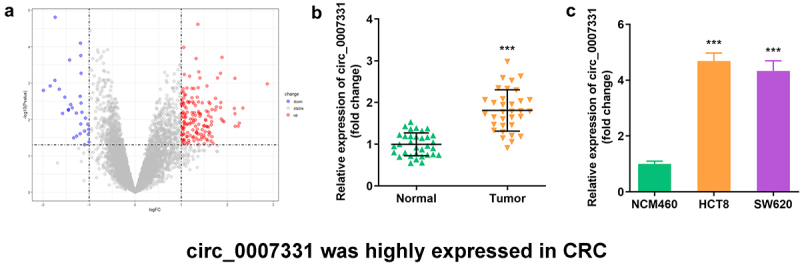

## 1. Introduction

Colorectal cancer (CRC) is a common gastrointestinal malignancy. According to statistics, CRC accounts for approximately 10% of all cancer-related deaths annually [1]. The incidence of CRC in males and females accounts for 9% and 8% of all cancers in men and women, respectively [2]. CRC is either asymptomatic or asymptomatic in its early stages, making it difficult to detect. The 5-year survival rate of patients with early diagnosis and surgery can be 90%, whereas that of patients with advanced CRC with metastasis is lower than 10% [3,4]. Hence, CRC is a serious threat to human health and requires further investigation.

Circular RNA (circRNA) is a type of special non-coding RNA that is transcribed by RNA polymerase II to form a covalently closed single-stranded circular RNA molecule without a 5’ cap and 3’ poly (A) tail [5]. CircRNAs are widely expressed in human tissues and organs, and their expression levels can reach more than 10 times the corresponding mRNA [6]. In addition, its circular structure makes it more stable and conserved than linear non-coding RNAs [7,8]. Moreover, circRNAs exhibit specific expression patterns in different cells [9]. These special features allow circRNAs to modulate gene expression at the transcriptional and post-transcriptional levels [10]. Chen et al. [11] identified 10,245 differentially expressed circRNAs in colorectal cancer expression profiles, among which 6,264 were upregulated. In addition, hsa_circ_001569 was found to increase the proliferation and invasion of tumor cells in different CRC cell lines [12]. circ_BANP can promote the proliferation of colorectal tumor cells through the PI3K/AKT pathway [13], indicating that circRNAs play an important regulatory role in CRC. circ_0007331 is a circular RNA located on chromosome 3, which is reported to be related to colon cancer [14], but its mechanism in CRC remains to be further clarified.

The high-mobility group (HMG) is a class of small non-histone proteins abundant in the nucleus, among which HMGA2 is highly expressed in malignant tumors [15]. The HMGA2 gene, 200 kb in length, is located at 12q13-15 [16] and is widely expressed during embryonic development, but is expressed at low levels in adult tissues [17]. HMGA2 was reported to take part in the regulation of tumor development. For instance, lncRNA HIT000218960 promotes the proliferation and migration of gastric cancer cells by overexpressing HMGA2 [18], and miRNA let-7 affects the progression of esophageal squamous cell carcinoma by negatively regulating HMGA2 [19]. HMGA2 is highly expressed [20]. Silencing HMGA2 can cause tumor cell apoptosis by arresting cell growth in the G2/M phase [21].

Therefore, this study aimed to explore the function and regulation of circ_0007331 in CRC cells. We hypothesized that circ_0007331 regulating the proliferation, migration and invasion via targeting the miR-205-5p/HMGA2 axis.

## 2. Materials and methods

### 2.1 Bioinformatic analysis

A microarray profile GPL19978 related to CRC was downloaded from the Gene Expression Omnibus (GEO) database (http://www.ncbi.nlm.nih.gov/geo). All differentially expressed genes (DEGs) of osteoporotic group (n = 3) and non-osteoporotic group (n = 3) were identified and then screened under the standard od P < 0.05 and |log2FC| ≥ 1.5. Additionally, the target miRNA of circ_0007331 was predicated in Starbase online database (http://starbase.sysu.edu.cn/). And the target gene of miR-205-5p was predicated by targetscan online database (http://www.targetscan.org/vert_71/).

### 2.2 Patients

CRC samples and adjacent non-tumor tissue samples were collected from CRC patients (n = 33) at Yuebei People’s Hospital from 1 January 2019, to 1 November 2020.

The study was approved by the Yuebei People’s Hospital. All the participants signed an informed consent form.

### 2.3 Cell culture

HCT 8 as well as SW620 cells (ATCC, Manassas, VA) were cultivated in DMEM (Thermo Fisher Scientific, USA). 10% FBS (Gibco, Waltham) and 1% penicillin/streptomycin (Gibco, Waltham) were added to the cell culture medium.

### 2.4 RT-qPCR

According to a previous study [22], RNA was extracted from HCT 8 and SW620 cells using a commercially available kit (Takara, Japan). Then, cDNA was synthesized, and PCR was performed using a real-time PCR detection system (Bio-Rad, USA). The primer sequences used were as follows.

circ_0007331: F: 5ʹ-GAATGGGATTCGAGACCTG-3ʹ, R: 5ʹ-TTCTTCCAAAGCTGCCTGT-3ʹ

miR-205-5p: F: 5’-TCCTTCATTCCACCGGAGTCTG-3’, R: 5’-GCG AGCACAGAATTAATACGAC-3’;

HMGA2: F: 5’-AGA UUGAGAUUGAAAGUGCCU-3’, R: 5’-GCA CUUUCAAUCUCAAUCUCU-3’

GAPDH: F: 5’-GAGTCCACTGGCGTCTTCAC-3’, R: 5’-ATCTT GAGGCTGTTGTCATACTTCT-3’.

### 2.5 Cell viability assay

According to a previous study [23], after resuspending the cells, they were inoculated into 96-well plates at 100 μl/well. Added to each well was 10 μl CCK8 reagent (AmyJet Technology Co., Ltd.), and it was cultured for 4 h at 37°C. A microplate reader (Nanjing DeTie Experimental Equipment Co., Ltd.) was used to measure the absorbance at 450 nm.

### 2.6 Colony formation assay

According to a previous study [24], HCT 8 and SW620 cells were inoculated into 6-well plates and cultured for 7 days with the culture medium refreshed every 2 days. Thereafter, HCT 8 as well as SW620 cells were stained by crystal violet (0.1%) for ten minutes. The colonies were observed under a microscope (Nikon, Tokyo, Japan).

### 2.7 Transwell assay

According to a previous study [25], 1 × 10 ^6^ of HCT 8 as well as SW620 cells were seeded into a 24-well upper chamber precoated with Matrigel (Corning Inc., USA). After 48 h, the upper chamber cells were removed, and the invading cells were fixed with methanol and stained with crystal violet. The cells were then examined under a microscope.

### 2.8 Dual luciferase reporter assay

According to a previous study [26], the binding sites of circ_0007331 and miR-205-5p were predicted using StarBase 3.0. After cultivation for 24 h, cells were lysed. A luciferase reporter assay kit (BioVision Tech Co., Ltd.) was used to analyze luciferase activity 48 h after co-transfection with miR-205-5p mimic/control as well as luciferase reporter vectors. Luciferase activity was determined using a commercial kit (Promega).

### 2.9 RNA pull-down

According to a previous study [27], the MagCapture RNA Pull Down Assay Kit (Whatman Co., Ltd.) was used for the RNA pull-down assay, according to the manufacturer’s protocols. The proteins were collected for mass spectrometry analysis.

### 2.10 Western blot

According to a previous study [28], protein extracts were loaded onto 10% SDS gel electrophoresis. Then The protein extracts were then transferred to a PVDF membrane (Millipore) and incubated with primary antibodies overnight at 4°C. One day later, the membranes were incubated with secondary antibodies at room temperature for two hours. Finally, the bands were captured using an enhanced chemiluminescence system (Thermo Fisher Scientific, Inc.).

### 2.11 Xenograft model assay

According to a previous study [29], twelve BALB/c nude mice (male, 4-week-old) were provided by Guangdong Medical Laboratory Animal Center (Guangdong, China). The animal experiment was under the approval of Yuebei People’s Hospital and met the demands of laboratory animal welfare and ethics. SW620 cells transfected with sh-nc or sh-circ_0007331 (2 × 10^6^) were injected into the back of mice and allowed to grow for 42 days to establish the Xenograft model. Finally, Xenograft volume and weight were examined.

### 2.12 Statistical analysis

Statistical analyses were performed using GraphPad Prism 7.0 (Graph-Pad Software, USA). Data are presented as mean ± standard deviation (SD). Two groups were analyzed using the Student’s t-test. The contrast between the two groups was analyzed by analysis of variance. Statistical significance was set at p < 0.05.

## 3. Results

Circ_0007331 expression was increased in CRC. The inhibition of circ_0007331 decreased the tumor cell viability, clone formation, migration, and invasion through the miR-205-5p/HMGA2 axis. Additionally, the inhibition of circ_0007331 suppressed the development of CRC in vivo.

### 3.1. circ_0007331 was highly expressed in CRC

**Figure 1.**
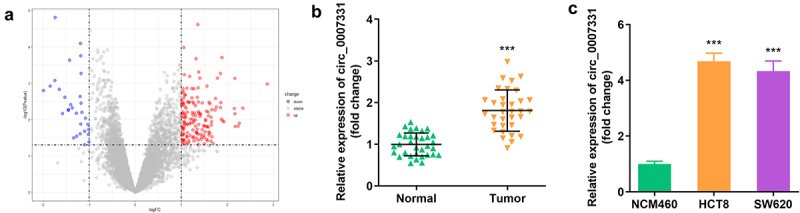
**Circ_0007331 was elevated in CRC**. (a) Volcano plots indicated the differentially expressed circRNAs between CRC and normal samples. (b) circ_0007331 expression in CRC patients. (c) circ_0007331 expression in HCT8 as well as SW620 cells.

We analyzed the microarray data of GPL19978 from the GEO database to select circRNAs related to CRC, and 156 circRNAs were significantly upregulated in CRC tissues, while 30 circRNAs were notably downregulated (Figure 1a). In addition, circ_0007331 was expressed more in CRC tissues, HCT8 cells, and SW620 cells (Figures 1b and 1c).

### 3.2 Knockdown of circ_0007331 reduced cell viability, colony formation, migration along with invasion of HCT8 and SW620 cells

**Figure 2.**
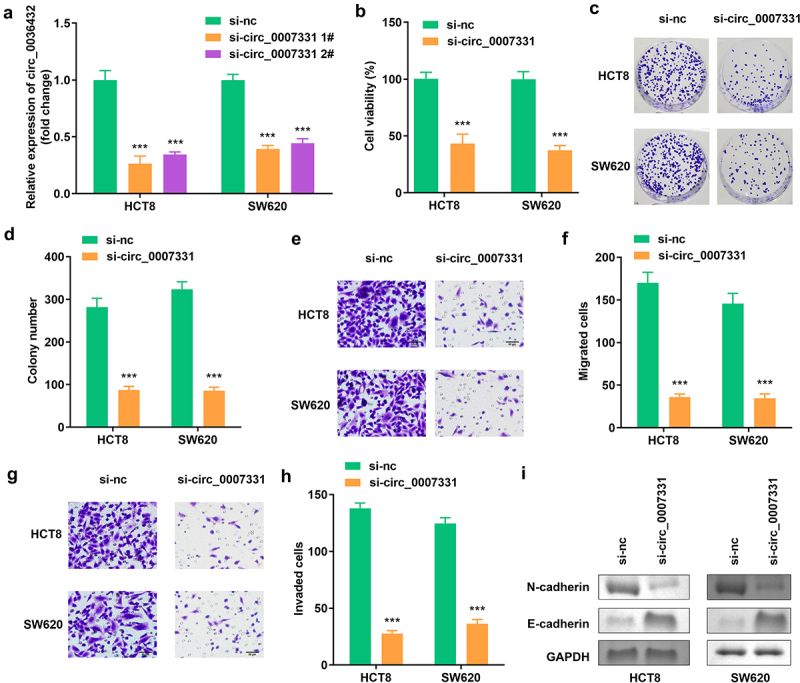
**Circ_0007331 knockdown declined aggressiveness of CRC cells**. (a) Expression of circ_0007331 in HCT8 as well as SW620 cells. (b) Cell viability of HCT8 as well as SW620 cells. (c, d) Clone formation of HCT8 as well as SW620 cells. (e, f) The migration of CRC cells. (g, h) The invasion of CRC cells. (i) The protein expression of N-cadherin and E-cadherin. *** P < 0.001 versus si-nc.

The expression of circ_0007331 was prominently decreased compared to the blank vector, indicating the success of transfection, and 1# was used in the following experiments (Figure 2a). Knockdown of circ_0007331 observably reduced the viability and colony formation of HCT8 and SW620 cells (Figure 2b-2d). Moreover, the migration and invasion of HCT8 and SW620 cells were reduced after circ_0007331 knockdown (Figure 2e-2h), while the protein expression of N-cadherin decreased, whereas that of E-cadherin increased. (Figure 2i).

### 3.3. circ_0007331 directly targeted miR-205-5p

**Figure 3.**
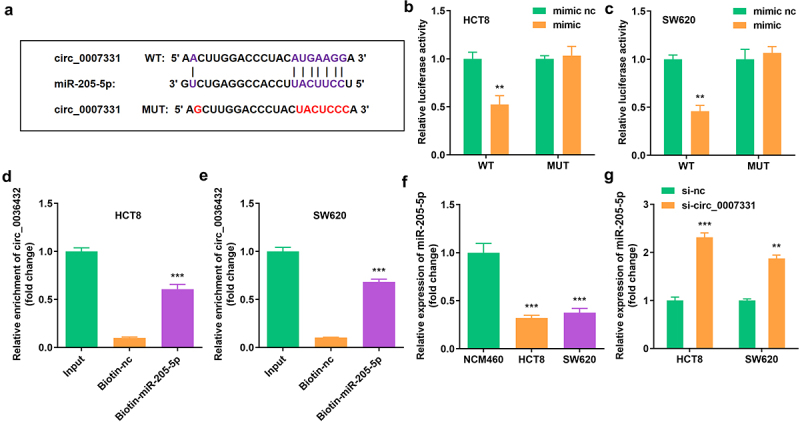
**Circ_0007331 directly targeted miR-205-5p** (a) The binding sites between circ_0007331 and miR-205-5p. (b, c) The luciferase activity of HCT8 and SW620 cells. (d, e) The interaction between circ_0007331 and miR-205-5p. (f) miR-205-5p expression in HCT8 and SW620 cells. (g) miR-205-5p expression in cells with circ_0007331 knockdown. **P < 0.01, *** P < 0.001 versus control.

The binding region of miR-205-5p and circ_0007331 was obtained in Starbase online database (http://starbase.sysu.edu.cn/) (Figure 3a), which was verified using a dual luciferase reporter assay along with an RNA pull-down assay (Figure 3b-3e). miR-205-5p expression was markedly suppressed in HCT8 and SW620 cells (figure 3f), whereas circ_0007331 knockdown notably upregulated the expression of miR-205-5p in HCT8 and SW620 cells (Figure 3g).

### 3.4 Inhibition of miR-205-5p abrogated the effects of depletion of circ_0007331 in HCT8 and SW620 cells

**Figure 4.**
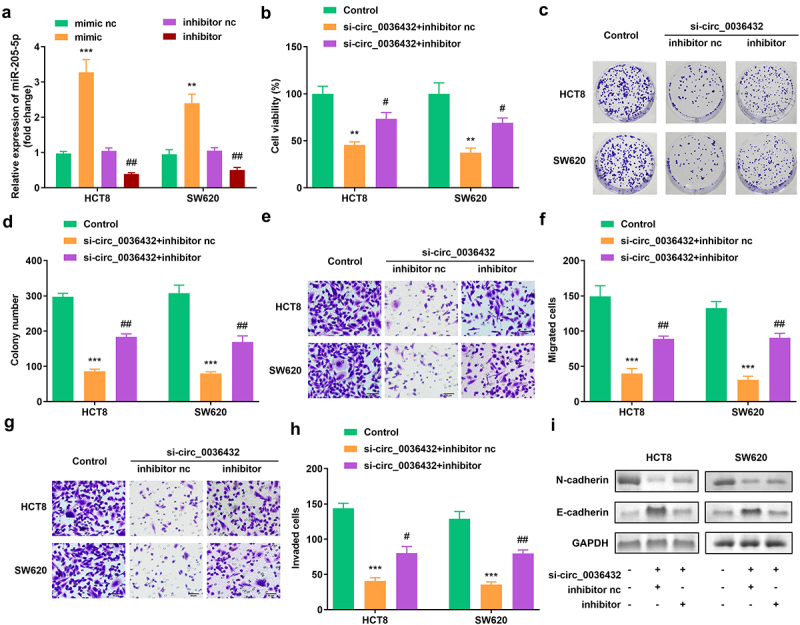
**miR-205-5p inhibition reversed the effects of circ_0007331 knockdown**. (a) Expression of circ_0007331 in HCT8 as well as SW620 cells. (b) Cell viability of HCT8 as well as SW620 cells. (c, d) Clone formation of HCT8 as well as SW620 cells. (e, f) The migration of CRC cells. (g, h) The invasion of CRC cells. (i) The protein expression of N-cadherin and E-cadherin. #P < 0.05, ##P < 0.01, **P < 0.01, *** P < 0.001 versus control.

Figure 4a shows that, compared with the blank vector, miR-205-5p expression was increased by the mimic and downregulated by the inhibitor. miR-205-5p inhibition remarkably improved the viability and colony formation of HCT8 and SW620 cells, which reversed the decrease caused by circ_0007331 knockdown (Figure 4b-4d). In addition, the depletion of miR-205-5p enhanced the migration and invasion of HCT8 and SW620 cells (Figure 4e-4h). In addition, the protein expression of N-cadherin and E-cadherin was reversed after the inhibition of miR-205-5p(Figure 4i).

### 3.5. miR-205-5p directly targeted HMGA2

**Figure 5.**
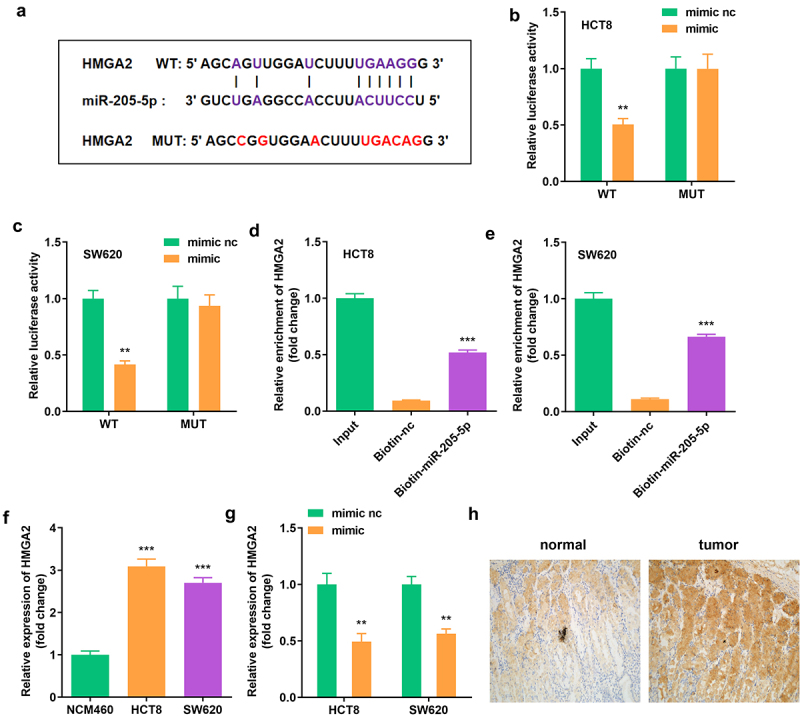
**miR-205-5p directly targeted HMGA2** (a) The binding sites between HMGA2 and miR-205-5p. (b, c) The luciferase activity of HCT8 and SW620 cells. (d, e) The interaction between HMGA2 as well as miR-205-5p. (f) Expression of HMGA2 in HCT8 and SW620 cells. (g) Expression of HMGA2 in cells with miR-205-5p overexpression. **P < 0.01, *** P < 0.001 versus control.

**Figure 6.**
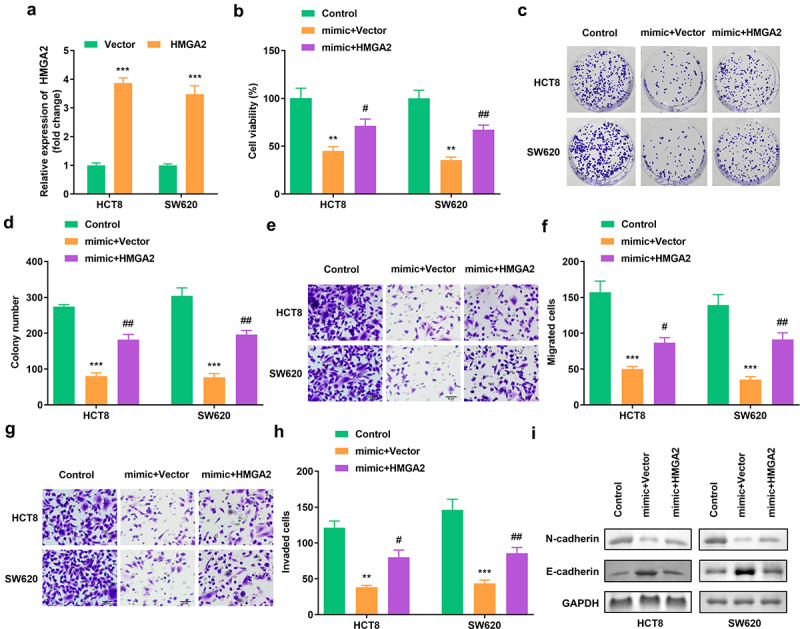
**Elevated HMGA2 neutralized the function of miR-205-5p on cell viability, colony formation, migration as well as invasion** (a) HMGA2 expression in HCT8 as well as SW620 cells. (b) Cell viability of HCT8 as well as SW620 cells. (c, d) Clone formation of HCT8 as well as SW620 cells. (e, f) The migration of CRC cells. (g, h) The invasion of CRC cells. (i) The protein expression of N-cadherin and E-cadherin. #P < 0.05, ##P < 0.01, **P < 0.01, *** P < 0.001 versus control.

To determine the regulatory axis, including circ_0007331 and miR-205-5p, TargetScan (http://www.targetscan.org/vert_71/) was used to predict miR-205-5p’s target gene; HMGA2 was found to be the regulatory axis (Figure 5a). Dual luciferase reporter assay and RNA pull-down assay were performed to confirm the targeting interaction of HMGA2 and miR-205-5p (Figure 5b-5e). figure 5f showed that HMGA2 was expressed highly in HCT8 and SW620 cells. Moreover, high miR-205-5p expression decreased HMGA2 expression in HCT8 and SW620 cells (Figure 6e).

### 3.6 Elevated HMGA2 neutralized the function of miR-205-5p on cell viability, colony formation, migration as well as invasion

A notable increase in HMGA2 expression resulted in a successful transfection (Figure 6a). HMGA2 overexpression rescued cell viability and colony formation (Figure 6b-6d). The migration and invasion of CRC cells was also enhanced after HMGA2 overexpression (Figure 6e-6h). Furthermore, the protein expression of N-cadherin was increased, whereas that of E-cadherin was decreased. (Figure 6i).

### 3.7 Depletion of circ_0007331 suppressed tumor growth in vivo

**Figure 7.**
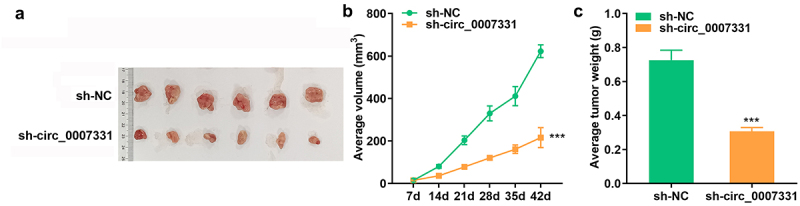
**Depletion of circ_0007331 suppressed tumor growth in vivo** representative images (a), tumor volume (b) and tumor weight (c) of tumors derived from xenograft models. *** P < 0.001 versus sh-NC.graphical abstract.

To further confirm the role of circ_0007331 in CRC, the xenograft tumor model was established and the tumor volume and weight were analyzed. The results showed that the volume (Figure 7a-b) and weight (Figure 7c) of tumors in sh-circ_0007331 group were significantly decreased compared with control group.

## 4. Discussion

CRC is a common malignant digestive tract tumor with high morbidity as well as mortality [30], and its incidence has increased rapidly in recent years [31,32]. This study reveals that the dysregulated circ_0007331 is connected with the occurrence and development of CRC. circ_0007331 knockdown reduced the activity and proliferation of tumor cells as well as migration and invasion through the circ_0007331/miR-205-5p/HMGA2 axis. To our knowledge, this is the first study to investigate the regulatory role of circ_0007331 in CRC development.

Circular RNAs are involved in various malignant tumors by inducing angiogenesis, and promoting tumor immune escape and the formation of an inflammatory microenvironment [33]. For example, circ_0078710 regulates miR-31 to accelerate the proliferation and migration of liver cancer cells [34], while circ_0014717 affects the tumor cell cycle and suppresses the development of CRC by upregulating the expression of P16 protein [35]. In this study, circ_0007331 levels increased in CRC. The activity, proliferation, migration, and invasion abilities of tumor cells were weakened after the knockdown of circ_0007331, suggesting that circ_0007331 plays a regulatory role in CRC.

Given that circRNAs can act as ‘ sponges ‘ to regulate miRNAs, we predicted that circ_0007331 targets miR-250-5p. It has been reported that miR-250-5p is a regulatory factor in various cancers, such as targeting ZEB1 to inhibit the aggressiveness of prostate cancer cells [36], promoting the development of colorectal cancer by targeting VEGFA [37], and suppressing the vitality and invasion of ovarian cancer cells by promoting apoptosis [38]. Here, miR-250-5p was less expressed in CRC but increased in tumor cells with circ_0007331 knockdown. In addition, inhibition of miR-250-5p can rescue the effect of circ_0007331 knockdown on cells, indicating that miR-250-5p is an important part of the circ_0007331 regulatory axis.

HMGA2 is a target of miR-250-5p. Studies have shown that high HMGA2 expression is linked to the occurrence, development, and prognosis of malignant tumors of the digestive tract [39], such as enhancing the invasion of gastric cancer by promoting epithelial-mesenchymal transition [40] and increasing the proliferation and differentiation of esophageal cancer cells [41]. This study also verified that increased HMGA2 expression enhances the aggressive behavior of CRC cells by modulating circ_0007331.

## 5. Conclusion

In conclusion, circ_0007331 expression is increased in CRC. The inhibition of circ_0007331 increased tumor cell viability, clone formation, migration, and invasion through the miR-205-5p/HMGA2 axis. These findings may provide potential therapeutic targets for CRC treatment.

## Data Availability

Not applicable.
